# Preparation of Novel Poly(hydroxyethyl methacrylate-*co*glycidyl methacrylate)-Grafted Core-Shell Magnetic Chitosan Microspheres and Immobilization of Lactase

**DOI:** 10.3390/ijms140612073

**Published:** 2013-06-06

**Authors:** Wei Zhao, Rui-Jin Yang, Ting-Ting Qian, Xiao Hua, Wen-Bin Zhang, Wendy Katiyo

**Affiliations:** 1 State Key Laboratory of Food Science and Technology, Jiangnan University, No. 1800 Lihu Road, Wuxi 214122, China; E-Mails: zhaow@jiangnan.edu.cn (W.Z.); shawaxe@gmail.com (X.H.); wbzhang@jiangnan.edu.cn (W.-B.Z.); wendykandy@yahoo.com (W.K.); 2 School of Food Science and Technology, Jiangnan University, No. 1800 Lihu Road, Wuxi 214122, China; E-Mail: tingt_qian@126.com

**Keywords:** magnetic chitosan microspheres, reversed-phase suspension polymerization, immobilization, lactase

## Abstract

Poly(hydroxyethyl methacrylate-*co*-glycidyl methacrylate)-grafted magnetic chitosan microspheres (HG-MCM) were prepared using reversed-phase suspension polymerization method. The HG-MCM presented a core-shell structure and regular spherical shape with poly(hydroxyethyl methacrylate-*co*-glycidyl methacrylate) grafted onto the chitosan layer coating the Fe_3_O_4_ cores. The average diameter of the magnetic microspheres was 10.67 μm, within a narrow size distribution of 6.6–17.4 μm. The saturation magnetization and retentivity of the magnetic microspheres were 7.0033 emu/g and 0.6273 emu/g, respectively. The application of HG-MCM in immobilization of lactase showed that the immobilized enzyme presented higher storage, pH and thermal stability compared to the free enzyme. This indicates that HG-MCM have potential applications in bio-macromolecule immobilization.

## 1. Introduction

A variety of polymeric support materials including natural (chitosan, agarose, and cellulose derivatives) and synthetic (nylon, polysiloxane/polyaniline, polyvinylalcohol, and polyacrylic polymers) have been prepared in various geometric forms and used for the immobilization of enzymes [1–6]. Recently, magnetic carrier technology has become popular for enzyme, protein and cell separation and immobilization [7,8]. When used as the support material, magnetic carriers can be quickly separated from the reaction medium and effectively controlled by applying a magnetic field. Thus the catalytic efficiency and stability properties of enzymes are greatly improved [7]. It was observed that alternating magnetic fields induce rotational and vibrational movements in polymeric beads exhibiting magnetically anisotropic properties. In an enzyme reactor, these magnetic phenomena could be used for preventing product film formation around the enzyme-magnetic beads [7,8].

There has been wide-spread interest in polymer-based magnetic microspheres because of their excellent properties such as biocompatibility, biodegradability, non-toxicity and abundant functional groups [9–11]. Chitosan is a type of biodegradable polysaccharide with abundant –NH_2_ and –OH groups, and this is desirable in its application as magnetic support material [12,13]. Magnetic chitosan microspheres (MCM), synthesized using glutaraldehyde as the cross-linking reagent, have been developed for enzyme immobilization [12,13]. Nevertheless, a great deal of research has focused on improving the physical and chemical properties of MCM via chemical modification on chitosan [14–17].

Polymer membranes were well functionalized by synthetic monomers having reactive functional groups (*i.e.*, amine, hydroxyl, carboxyl, and epoxy groups), such as glycidyl methacrylate (GMA), ethylene glycol dimethacrylate (EGDMA), methyl methacrylate (MMA) and 2-hydroxyethtyl methacrylate (HEMA) [18,19]. To the best of our knowledge, studies of functionalized magnetic chitosan microspheres by these monomers have rarely been reported. GMA is an excellent monomer with a reactive epoxy group, which can be directly coupled with a biological molecule via ring opening reactions or further modified into different functional groups [18]. HEMA can form long spacer arms that provide more reactive sites and reduce steric hindrance when binding. Moreover, HEMA is able to create a hydrophilic micro-environment at MCM surface [18]. In this study, the poly (HEMA-*co*- GMA)-grafted MCM (HG-MCM) was prepared using reversed-phase suspension polymerization method.

Furthermore, lactase was selected as a model enzyme for immobilization using the prepared HG-MCM. Lactase, which hydrolyses lactose into its monomers has important applications in the food processing industry [20]. Although most industries still hydrolyze lactose using free enzymes, the immobilization of lactase is an area of great interest because of its potential benefits [20]. The use of immobilization technology is of significance from an economic point of view. It makes reutilization of the enzyme and continuous operation possible, and also precludes the need to separate the enzymes and impurities from the reaction system following processing. In addition, it also helps to improve the enzyme stability. Nowadays, immobilized lactase is being used in lactose hydrolysis of milk/whey and has been tested for the production of galacto-oligosaccharides [20]. There have been some reports about the immobilization of lactase (*Kluyveromyces lactis*) using various materials. Lactase has been immobilized on graphite surface [21], silica and agarose [22], cotton fabric [23], and polymeric membrane surfaces [24]. Glutaraldehyde-activated chitosan was used for immobilization of lactase in a packed-bed reactor for the continuous hydrolysis of lactose. Almost complete lactose hydrolysis was achieved for both milk whey and lactose solution at 37 °C at flow rates up to 2.6 mL/min [25]. In our previous study [26], the alginate-gelatin-calcium phosphate hybrid carrier, which was the alginate capsule covered with gelatin film and calcified shell, was prepared through a facile biomimetic mineralization process, and further utilized to immobilize lactase. The immobilization efficiency achieved 83.2% and the relative activity of immobilized lactase was more than 60% after the 20th cycle and about 90% after 30 days of storage. In this study, reversed-phased suspension methodology was applied for the preparation of poly(HEMA-*co*-GMA)-grafted core-shell MCM. Chitosan properties could be improved by graft polymerization. Moreover, the polymeric matrix could protect the magnetic nanoparticles against agglomeration and adds the specific functional groups required for immobilization. Lactase was then immobilized on HG-MCM. The conditions for immobilization of the enzyme and its characterization were studied systematically.

## 2. Results and Discussion

### 2.1. Preparation of Magnetic Fluids

**Figure 1:**
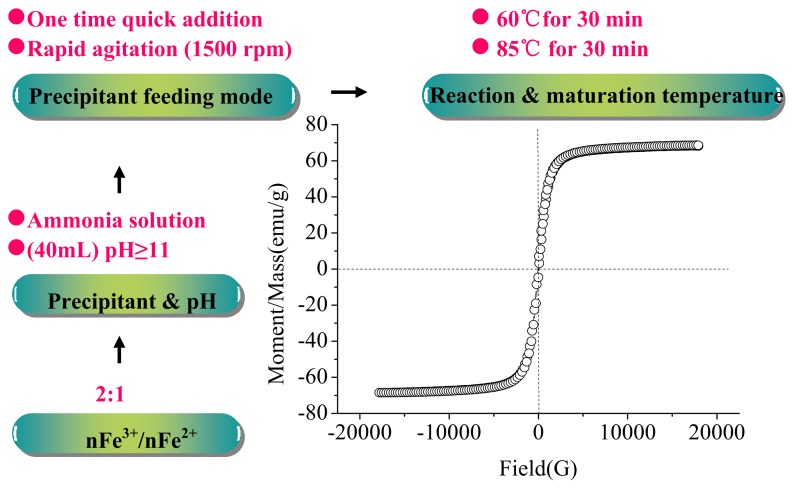
The optimized synthesis conditions and magnetic behavior of nanomagnetic particles (Fe_3_O_4_).

Superparamagnetic magnetite (Fe_3_O_4_) nanoparticles were prepared by chemical co-precipitation of Fe^2+^and Fe^3+^ salts with aqueous NH_3_. The suitable molar ratio of Fe^2+^/Fe^3+^, precipitant and pH of the reaction system, precipitant feeding mode and reaction, maturation temperature and time are important for preparation of Fe_3_O_4_ nanoparticles. These conditions are associated with the particle size, distribution and magnetic behavior of the nanomagnetic particles. The synthesis conditions of nanomagnetic particles (Fe_3_O_4_) were obtained and described in Figure 1. The prepared nanomagnetic particles (Fe_3_O_4_) had a mean particle size of 12 nm. X-ray diffractometer (XRD) analysis showed six characteristic peaks for Fe_3_O_4_, which were consistent with the database in JCPDS file (PDF No. 65-3107) (results not shown), indicating that the resultant nanoparticles were pure Fe_3_O_4_ with a spinel structure [18]. The saturation magnetization was 68.580 emu/g, as shown in Figure 1. The retentivity was 3.5007 emu/g, showing a superparamagnetic behavior.

### 2.2. Preparation of HG-MCM

Poly (HEMA-*co*-GMA)-grafted core–shell MCM were prepared by applying reversed-phase suspension methodology. Firstly, a stable dispersion of magnetite nanoparticles was dispersed in chitosan-acetic acid solution. Due to the electrostatic interaction between cationic chitosan and negatively charged Fe_3_O_4_ nanoparticles, the chitosan-coated nanoparticles could be obtained. This also improved stability of the magnetite nanoparticles in the acid solution [14]. Secondly, GMA and HEMA were added as the cross-linking and hardening agents for copolymerization and modification of magnetic chitosan microspheres. Finally, the monomers were polymerized by initiation with ammonium persulfate. As the polymer chains grew and eventually reached a molecular weight exceeding a critical value, they precipitated from solution on magnetite nanoparticles and aggregated to form colloidal particles. This resulted in formation of the HG-MCM. The grafted GMA and HEMA provided reactive epoxy groups and reduced steric hindrance during binding. They also created a hydrophilic micro-environment on the MCM surface [18], thus resulting in an increase in enzyme immobilization capacity and enzyme activity retainment.

**Figure 2:**
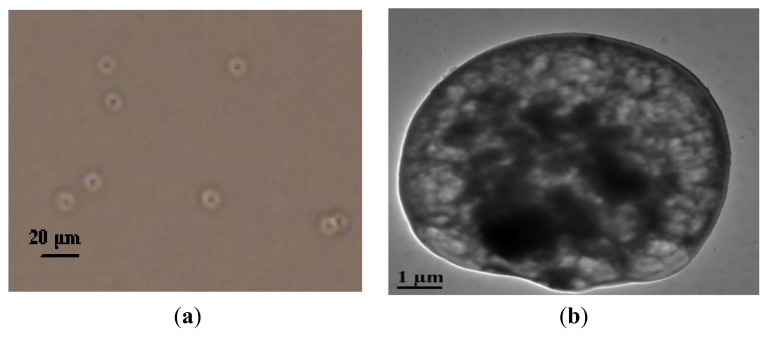

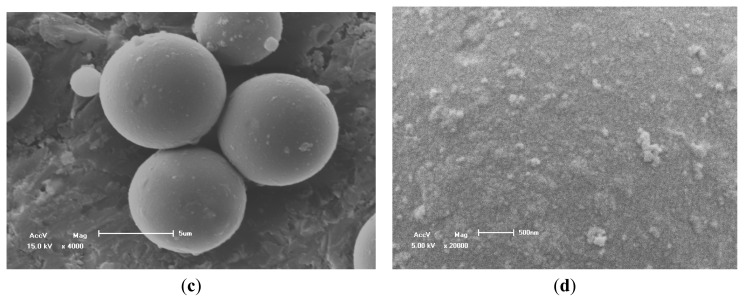
Morphology of HG-MCM: (**a**) An optical observation (magnification = 400); (**b**) TEM 144 image (magnification = 4000); SEM observation on HG-MCM: (**c**) Magnification = 4000 and (**d**) Magnification = 20,000.

From the optical observation (400×) on HG-MCM (Figure 2a), it can be clearly seen that the synthesized product presented a core-shell structure. An aggregated core, namely Fe_3_O_4_, was enclosed by a polymer shell. This structure was further confirmed by TEM characterization (Figure 2b), in which the black particles represented Fe_3_O_4_. From SEM observation (Figure 2c), it was clear that the synthesized materials were regular spheres with a smooth surface. In the 20000-fold magnified image (Figure 2d), a compact structure was seen on HG-MCM surface, which suggests that the polymerization took place at the interface of oil/water phases. The diameter of HG-MCM measured in SEM pictures was 5–10 μm.

**Figure 3:**
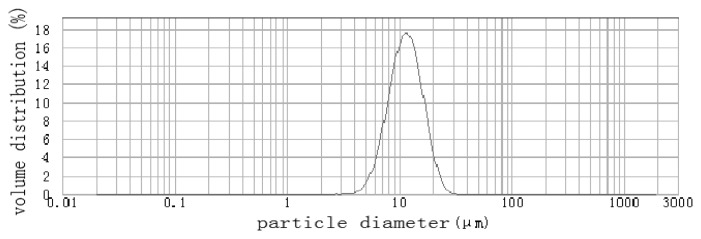
Size distribution of HG-MCM.

According to size analysis by dynamic light scattering (Figure 3), the average diameter of the beads was 10.67 μm, and this is 3–5 μm greater than that of SEM observation. This difference can be attributed to the swelling of chitosan in aqueous solution during size analysis. Moreover, HG-MCM have narrow size distribution, 6.6–17.4 μm, which reflects the homogeneous formation of a micro-template. The percentage of particles with diameter in the ranges 3.3–6.6 μm, 6.6–17.4 μm and 17.4–30 μm were 5.86%, 84.14% and 9.97%, respectively. The product has a small specific surface area of 0.562 m^2^/g, which is in good agreement with its spherical shape and smooth surface.

**Figure 4:**
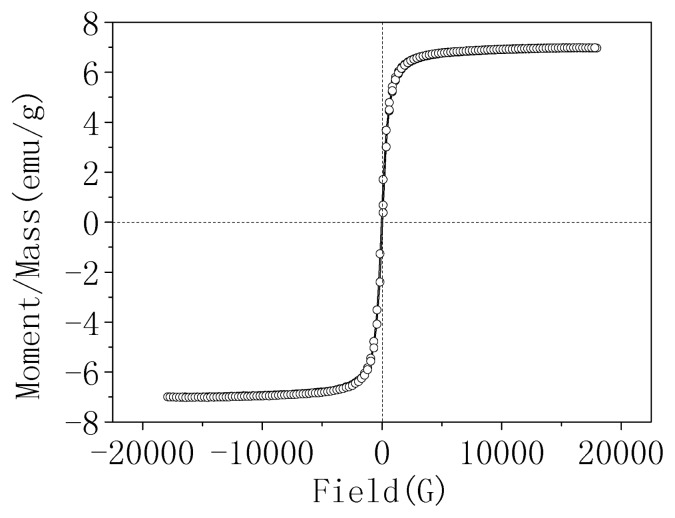
The magnetic behavior of HG-MCM.

The magnetic properties of HG-MCM were tested and shown in Figure 4. The experimental saturation magnetization and the retentivity of HG-MCM were 7.0033 emu/g and 0.62730 emu/g, respectively. It shall be noted that no coercive force was found in the hysteresis curve, which implies that the prepared materials had excellent paramagnetism. Actually, the prepared HG-MCM can be easily magnetized and reclaimed with an external magnet and dispersed quickly once the magnet is removed.

**Figure 5:**
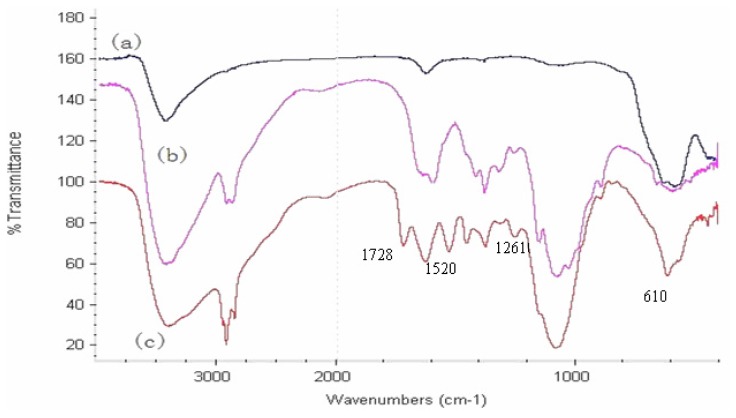
FTIR spectra of (**a**) magnetic fluid (**b**) chitosan (**c**) modifided magnetic chitosan microsphere.

A Fourier transform infrared (FTIR) spectrum of HG-MCM (Figure 5) shows a strong absorption at 610 cm^−1^ which can be attributed to the vibration of Fe–O bond [14]. The intense band that appears at 1728 cm^−1^ is attributed to C=O stretching frequency of conjugated ester group from GMA and HEMA [27]. The absorption at 1261 cm^−1^ is assigned as an epoxy group of GMA [28]. The FTIR spectrum of HG-MCM has some adsorption bands different from those of chitosan. The most important adsorption band at 1520 cm^−1^, representing N-H bending, is due to chitosan bonded to the poly(HEMA-*co*-GMA) [18].

**Figure 6:**
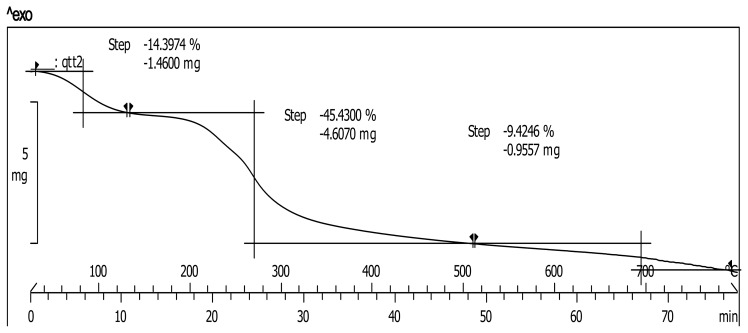
TGA of HG-MCM.

Thermogravimetric analysis (TGA) results of the HG-MCM are shown in Figure 6. For unbound Fe_3_O_4_, the TGA curve (DATA not shown) showed that the weight loss from 30 to 800 °C was about 0.7%. For HG-MCM, one peak below 150 °C caused by the loss of residual water resulted in a loss of 14.4%. Two other peaks appeared between 150–500 °C and 600–705 °C, showing weight losses of 45.43% and 9.42%, respectively. These were related to the degradation of chitosan and monomers. Beyond that, there was no significant weight change, implying the presence of only iron oxide. As a result, the amount of HG-MCM coated Fe_3_O_4_ was 30.70%.

### 2.3. Immobilization of Lactase on HG-MCM

**Figure 7:**
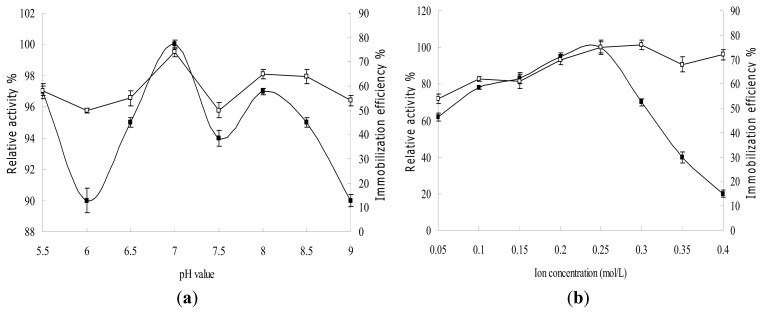

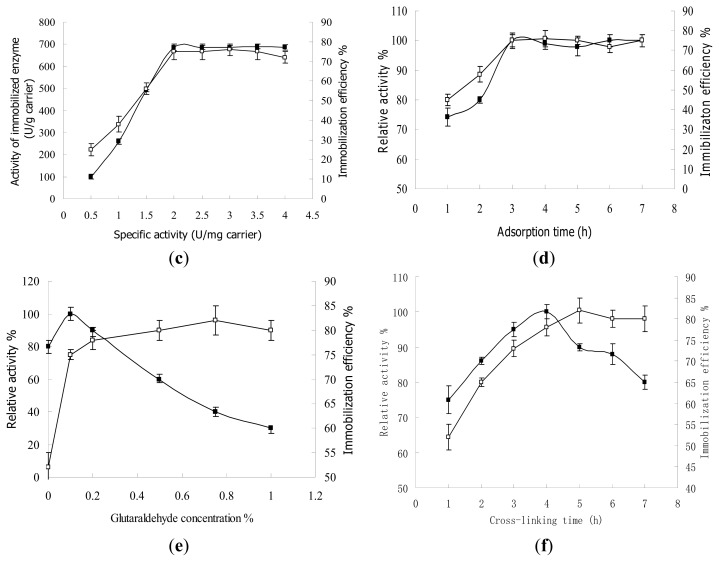
Effects of immobilization conditions on the immobilization efficiency (□) and relative activity of immobilized enzyme (■). pH (**a**) and ion concentration (**b**) of immobilization buffer, enzyme dosage (**c**) and adsorption time (**d**), glutaraldehyde concentration (**e**) and cross-linking time (**f**).

To determine the influence of pH and ion concentration of immobilization buffer, an experiment was performed under the following conditions: phosphate buffer pH (5.5–9) at a concentration of 0.1 mol/L or phosphate buffer concentration (0.05–0.4 mol/L) at pH 7.0. All other conditions were fixed; enzyme concentration (2 U/mg carrier), adsorption time (4 h), glutaraldehyde concentration (0.1%) and glutaraldehyde cross-linking time (4h). As shown in Figure 7a,b, the pH and ion concentration of immobilization buffer had significant influence (ρ < 0.05) on the relative activity and immobilization efficiency. The maximum relative activity and immobilization efficiency was achieved at phosphate buffer pH 7 and ion concentration 0.25 mol/L. Moreover, at pH 5.5 and 8, higher values of relative activity and immobilization efficiency were also observed. This might have been caused by the HG-MCM adsorption of enzyme which can be achieved through both physical adsorption and binding of epoxy groups. Therefore, the influence of pH on the binding of these epoxy groups might have played a main role at these pH zones.

The influence of enzyme dosage and adsorption time was investigated under the following conditions: enzyme concentration (0.5–4 U/mg carrier) with adsorption time (4 h) or enzyme concentration (2 U/mg carrier) with adsorption time (1–7 h). All other conditions were fixed; phosphate buffer pH (7) at concentration 0.25 mol/L, glutaraldehyde concentration (0.1%) and glutaraldehyde cross-linking time (4 h). Figure 7c,d shows that the relative activity and immobilization efficiency firstly increased with increase in enzyme concentration and adsorption time (*p* < 0.05), and reached its climax at an enzyme dosage of 2 U/mg carrier and adsorption time of 3 h. It then remained constant, indicating that the enzyme immobilization had already reached its maximal and the carrier was saturated with enzyme. The maximal enzymatic activity reached 685 U/(g carrier) at an enzyme dosage of 2 U/mg carrier and adsorption time of 3 h (Figure 7c) and the activity retention of the immobilized enzyme reached maximum, 48.18%. Different from other studies [29], the immobilization time had no significant influence (*p* > 0.05) on the immobilization efficiency and enzyme activity because the immobilization process was rapid. It could be that poly(HEMA-*co*-GMA) grafted onto the chitosan in this study provided more binding sites and formed a compact structure through polymerization. It therefore needed more time to achieve saturation for enzyme adsorption.

To establish the influence of glutaraldehyde concentration and cross-linking time, an experiment was performed under the following conditions: glutaraldehyde concentration (0%–1%) with cross-linking time (4 h) or glutaraldehyde concentration (0.1%) with cross-linking time (1–7 h). All other conditions were fixed; phosphate buffer pH (7) at concentration of 0.25 mol/L, enzyme dosage 2 U/mg carrier and adsorption time 3 h. Figure 7e,f shows that the immobilization efficiency firstly increased with increase in glutaraldehyde concentration (*p* < 0.05) The maximum relative activity was achieved at a glutaraldehyde concentration of 0.1%. It then decreased significantly. Glutaraldehyde is both a cross-linking agent and denaturant for enzymes. Hence, when the glutaraldehyde concentration increased, the immobilization efficiency also increased. However, a higher glutaraldehyde concentration could denature the enzyme and decrease its relative activity (Figure 7e). In comparison with previous studies [29], the glutaraldehyde concentration used in this study is much lower. This is due to the more binding sites and formation of a compact structure through polymerization by poly(HEMA-*co*-GMA). It is beneficial for maintainance of enzyme activity. Figure 7f shows that immobilization efficiency increased with prolonged cross-linking time (*p* < 0.05). The relative activity increased to a maximum at cross-linking time of 4 h, and then dramatically declined. This phenomenon was related to over cross-linking. Over cross-linked HG-MCM could block the contact of enzyme and substrate.

Based on the above results, the optimized conditions for immobilization of lactase on HG-MCM were as follows: phosphate buffer pH 7 at concentration of 0.25 mol/L, enzyme dosage 2 U/mg carrier and adsorption time 3 h, glutaraldehyde concentration 0.1% with cross-linking time 4 h. Under these conditions, 685 U/(g carrier) of immobilized enzyme activity, 48.18% of activity retention and 81% of immobilization efficiency [*i.e.*, the protein loading 319 mg/g carrier (31.9%)] were obtained. Macro-, micro- and nanosized chitosan particles were prepared as immobilization carriers for lactase (*K. lactis*) by precipitation, emulsion cross-linking and ionic gelation methods, respectively [30]. The protein loading for macro- chitosan particles (mean particle size 3200 μs) was relatively high (60%), however, the specific activity was very low. The microspheres and nanoparticles obtained with sodium tripolyphosphate and cross-linked with glutaraldehyde showed lower protein loading (23%). Although the largest amount of protein (38%) was bound by the nanoparticles obtained with sodium sulphate and cross-linked with glutaraldehyde, the storage stabilities of immobilized enzymes were the lowest. More than 60% activity was lost after 3 weeks in 0.02 mol/L potassium phosphate buffer (pH 7) at 4 °C. In our previous study [26], similar immobilization effects were obtained employing the alginate-gelatin-calcium phosphate hybrid carrier. However, magnetic microspheres used in this study possess superior catalytic and separation properties.

### 2.4. Properties of Immobilized Lactase on HG-MCM

#### 2.4.1. pH and Temperature Stability

**Figure 8:**
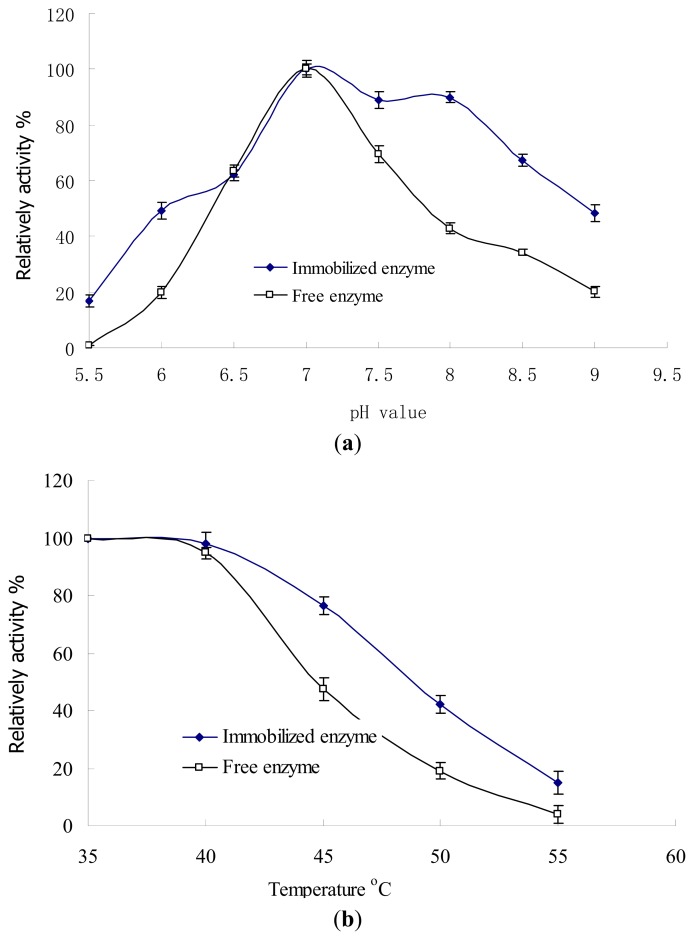
Effect of pH (**a**) and temperature (**b**) on the activities of free and immobilized enzyme.

The pH and temperature dependence of the free and immobilized enzyme activities was investigated (Figure 8). In comparison with free lactase, the pH and temperature stability of immobilized enzyme significantly improved (*p* < 0.05). Figure 8a shows that both the free and immobilized enzymes exhibited maximal enzymatic activity at pH 7.0. When pH was between 6.5 and 7.0, they showed almost the same activity. However, increase in pH to 7.5–9.0 or decrease to 6.0–5.5, resulted in the immobilized enzyme activity being significantly higher than that of the free enzyme. This demonstrates the enhanced stability of immobilized enzyme in alkaline and acidic conditions. At pH 9, the relative activity of free enzyme was 20%, while that of the immobilized enzyme was 54%. Figure 8b illustrates the effect of temperature on the native and the immobilized enzyme activities. The results show that immobilized enzyme was less sensitive to the change in temperature from 35 to 55 °C (*p* < 0.05). The relative activity of free enzyme decreased to 20% at 50 °C, while the immobilized enzyme maintained almost 50% of its relative activity. The enhanced pH and temperature stability might be caused by multipoint covalent immobilization. The configuration of enzyme is fixed on the surface of carriers, hence its tolerability to pH and temperature variance in the environment is increased [29]. Moreover, microenvironment of the immobilized enzyme in the carrier could have been buffered [29], which also improves the enzyme stability. Consequently, the immobilized enzyme could work in harsh environmental conditions with less activity loss compared to the free enzyme.

#### 2.4.2. Kinetic Properties

The catalytic constant (*K*_cat_) and kinetics parameter *K*_m_ for free and immobilized enzyme was determined using ONPG as the substrate. In enzymes, Michaelis-Menten kinetic behavior was observed. The *K*_cat_ (1176.2/s) for the immobilized lactase was similar with that (1182.9/s) for the free one. The *K*_m_ value of immobilized lactase (49.36 mmol/L) was higher than that of free enzyme (25.4 mmol/L) and lactase immobilized on MCM without the grafted layer (41.7 mmol/L), which means immobilized lactase had lower affinity towards the substrate. This is in agreement with other investigators who reported significant decrease in affinity in immobilized catalysts [7]. The increase in *K*_m_ might be caused by steric hindrance of the active site by the support, loss of enzyme flexibility necessary for substrate binding, or diffusion limitation of substrate and products because of the support [6]. It is worth noting that the study using magnetic chitosan microspheres without grafting showed that the *K*_m_ value of immobilized enzyme was several times that of its free counterpart. In the present study, the ratio of *K*_m_ value of immobilized and enzyme was only 1.9. This indicates that poly (HEMA-*co*-GMA) grafted onto the chitosan successfully reduced steric hindrance during binding.

#### 2.4.3. Storage Stability and Reusability

Storage stability and reusability is one of the most important advantages of immobilization. HG-MCM immobilized lactase was stable for over 12 months under dried condition at 4 °C with no loss in its activity. The HG-MCM immobilized lactase was also stored in phosphate buffer (0.1 mol/L, pH 7.0) at room temperature for 60 days. The immobilized enzyme lost less than 10% of its initial activity during storage.

**Figure 9:**
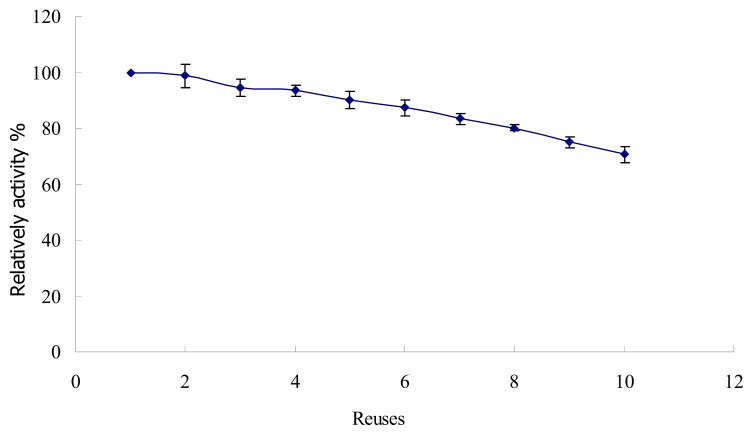
Reusability of immobilized enzyme.

With repeated reuse, the strength of binding between the matrix and enzyme is weakened and frequent encountering of substrate in the active site causes its distortion, leading to loss in activity [31]. However, as shown in Figure 9, immobilized lactase can be reused over 10 times with only 20% loss in its activity, which implies that the enzyme is strongly bound to HG-MCM.

## 3. Experimental Section

### 3.1. Materials

The monomers, GMA and HEMA, were purchased from Ciba Speciality Chemicals Lts (Guanzhou, China). They were distilled under reduced pressure prior to use. Ferric chloride hexahydrate (FeCl_3_·6H_2_O, >99%), ferrous sulfate (FeSO_4_·7H_2_O, >99%) and glutaraldehyde (GA, 25% *v*/*v* in aqueous solution) were obtained from Sinopharm Chemical Reagent Co. Ltd. (Shanghai, China). Chitosan, with deacetylation degree (DD) of 90% and molecular weight (M^w^) of 200 kD, was purchased from Jinke Biochemical Company (Taizhou, China). Lactase DSM Maxilact^®^ 5000 from *Kluyveromyces lactis* was a gift from DSM (Beijing, China). All the other chemicals used were of analytical grade.

### 3.2. Preparation of Magnetic Fluids

Fe_3_O_4_ nanoparticles were prepared by co-precipitating Fe^2+^ (FeSO_4_·7H_2_O) and Fe^3+^ (FeCl_3_·6H_2_O) ions in ammonia solution with N_2_ as the protective gas. The equation is as follows:

$$8{OH}^{-}+{Fe}^{2+}+2{Fe}^{3+}=Fe_{3}O_{4}↓+4H_{2}O$$

The optimized conditions for the preparation of magnetic fluids were investigated: the molar ratio of *n*Fe^3+^/*n*Fe^2+^ (3:1, 2:1, 1:1), precipitant & pH (ammonia solution, pH 9–12), precipitant feeding mode (one time quick addition or several times slow addition, agitation speed 100–2000 rpm), and reaction & maturation temperature and time (50–90 °C for 20–60 min). Based on our preliminary study, the synthesis conditions of nanomagnetic particles (Fe_3_O_4_) were obtained. A mixture of FeSO_4_·7H_2_O (5.84 g) and FeCl_3_·6H_2_O (11.35 g) was agitated in 120 mL deionized water under N_2_ atmosphere. Precipitates were formed by addition of 40 mL ammonia solution (25%) at 60 °C. The mixture was held for 30 min intervals, firstly at 60 °C and then at 85 °C, before cooling to room temperature. Products were separated using a magnet and fully washed with deionized water to remove excess ammonia. Finally, the product was vacuum dried at room temperature for further use.

### 3.3. Preparation of HG-MCM

Briefly, 20 mL acetic acid solution (1.5%, *w*/*v*) of chitosan (3%, *w*/*v*) and magnetic fluids (10% of chitosan, *w*/*w*) was added dropwise into a mixture of paraffin oil (80 mL) and Span 80 (6 mL) at 40 °C under N_2_ atmosphere. After stirring for 30 min, the temperature was increased to 60 °C. A mixture of HEMA (0.2 mL), GMA (0.4 mL), ethanol (0.3 mL) and deionized water (0.3 mL) and the initiator (0.1 g ammonium persulfate in 1.5 mL H_2_O) were introduced alternately every 10 min. After stirring for 4 h under N_2_ atmosphere, the mixture was held at 60 °C for 30 min and cooled to ambient temperature. The reaction of polymer grafting into chitosan is as follows [18,32,33]:

**Figure:**
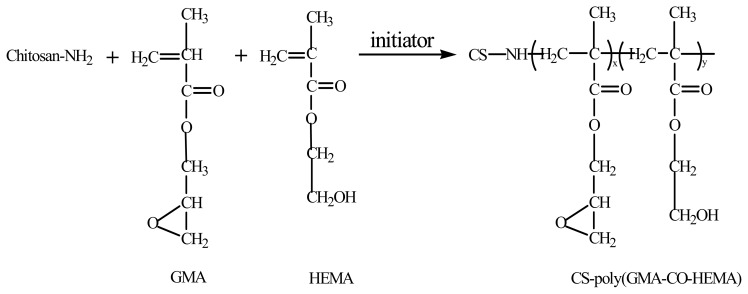


The products were collected with a magnet, washed consecutively with isopropyl alcohol, cyclohexane, acetone and deionized water and stored in vacuum.

### 3.4. Characterization of Magnetic Fluids and HG-MCM

Morphology was analysed using an optical microscope (Olympus BX51, Tokyo, Japan), transmission electron microscope (JEOL, JEM-2100, Tokyo, Japan) and a scanning electron microscope (Shimadzu SSX-550, Tokyo, Japan). The specific surface was determined with a surface area and porosimetry analyzer (TriStar 3000, Norcross, GA, USA). FT-IR spectra was recorded on a Nicolet, Nexus 470 spectrometer. The size distribution analysis was performed using a laser particle size analyzer (Mastersizer 2000, Malvern, UK). Magnetic property of HG-MCM was tested with a vibrating-sample magnetometer (LakeShore 7407, Lake Shore, Westerville, OH, USA). XRD, (Thermo ARL X’TRA, Waltham, MA, USA) using a monochromatized X-ray beam with nickel-filtered Cu Kα radiation. Thermogravimetric analysis (TGA) was carried out on a TGA system (Mettler Toledo TGA/SDTA851, Columbus, OH, USA) in nitrogen atmosphere, at a heating rate of 10 °C/min with sample weight of 4–5 mg.

### 3.5. Immobilization of Lactase on HG-MCM

The immobilization of lactase on HG-MCM was carried out by swelling, adsorption, covalent binding with reactive functional groups, and glutaraldehyde cross-linking. Magnetic microspheres (1 g) were swollen in 50-mL phosphate buffer with different pH and concentrations at 30 °C. Different concentrations of lactase were added for immobilization. Glutaraldehyde was finally added as the cross-linking agent. In order to investigate the optimized immobilization conditions, the following selected variables and their levels were employed; phosphate buffer pH (5.5–9), ion concentration (0.05–0.5 mol/L), enzyme concentration (0.5–4 U/mg carrier), adsorption time (1–7 h), glutaraldehyde concentration (0%–1.0%) and glutaraldehyde cross-link time (1–7 h). At the end of immobilization, the magnetic nanoparticles bound with enzyme were collected and washed several times with phosphate buffer (pH 7, 0.1 mol/L). The results were converted to relative activities (percentage of the maximum activity obtained in that series). The supernatant was used for protein determination according to the reported method [29]. The protein content of enzyme was 45 mg/mL. The immobilization efficiency of protein was calculated by subtracting the protein recovered in the supernatant from the protein subjected to immobilization.

### 3.6. Assay of Lactase Activity

Lactase activity was measured according to the reported method [34]. The hydrolysis of *O*-nitrophenyl-β-d-galactopyranoside (ONPG), at 0.5 g/L in 50 mM potassium phosphate buffer containing 1 mmol/L MgCl_2_ (pH 6.5), was assayed at 40 °C, The samples were drawn (1 mL each time) at different time intervals. The reaction was stopped by adding 1 mL of 0.25 mol/L H_2_SO_4_ to 0.5 mL of sample. Afterwards, 1.5 mL of 1 mol/L NaCO_3_ was added to develop the yellow color due to the presence of *O*-nitrophenol (ONP). This color was measured spectrophotometrically at a wavelength of 420 nm. Maxilact^®^ 5000 expresses a lactase activity of 228 U/mL. One unit (U) of enzyme activity was defined as the amount of enzyme required to liberate 1 μmol of ONP per minute under the assay conditions. The activity of immobilized lactase was measured in a similar manner, except that magnetic nanoparticles were removed by magnetization before measurement.

### 3.7. The pH and Temperature Stability of Free and Immobilized Enzyme

The free and immobilized enzyme activities were determined after incubation in 20 mL phosphate buffer (0.1 mol/L, pH 7.0) for 24 h in the temperature range of 30–55 °C. The effect of pH on the activities of free and immobilized enzyme was investigated at 37 °C under different pH conditions (0.1 mol/L phosphate buffer at pH 5.0–9.0. Sodium hydroxide was used to adjust the pH to 9) for 24 h.

### 3.8. Kinetic Properties

The Michaelis constant (*K*_m_) and catalytic constant (*K*_cat_) were determined by measuring the initial reaction rates of free and immobilized lactase in different concentrations of ONPG (6.3, 12.6, 18.9, 27, and 31.5 μmol/L) as substrate at 37 °C. The Lineweaver-Burk plots were made and *K*_m_ and *K*_cat_ were calculated.

### 3.9. Reusability Assay

The reusability of immobilized lactase was performed as follows: immobilized enzyme was incubated with lactose (60%, *w*/*v*) at 40 °C for 2 h, and then its activity was assayed as mentioned above. After the activity assay, the enzyme was separated and washed three times with phosphate buffer (0.1 mol/L, pH 7.0), and fresh lactose solution was added to start the next round. The activity obtained each round was compared with that of the first run (first run activity defined as 100%).

### 3.10. Statistical Analysis

All measurements were carried out in triplicate. Data were subjected to one-way analysis of variance (ANOVA) followed by Duncan’s multiple range test to identify differences amongst the means at *p* < 0.05 using a SPSS software package (version 11.03, SPSS, Chicago, IL, USA).

## 4. Conclusions

This work reported the synthesis of a polymer-based magnetic material via reversed-phase suspension polymerization method. Regular spheres with core-shell structure and smooth surfaces were prepared in a water-in-oil template. A novel product, HG-MCM, with special magnetic properties, abundant functional groups and a narrow particle size distribution was produced and successfully applied in the immobilization of a model lactase enzyme. The properties of lactase were highly improved. Therefore, HG-MCM having abundant functional groups has potential applications in bio-macromolecule immobilization.
